# From tissue to blood: an integrated multi-omics signature identifies fibrogenesis and neutrophil activation as key drivers of ulcerative colitis severity

**DOI:** 10.3389/fimmu.2026.1884480

**Published:** 2026-07-21

**Authors:** E. Ozcariz, M. Ostaszewski, O. Lopata, L. Ciglar, A. Y. F. Li Yim, M. Pruijt, X. Wang, G. A. Vignolle, F. Tran, V. Satagopam, G. R. D’Haens, S. Schreiber, P. Rosenstiel, C. Nöhammer, K. Vierlinger

**Affiliations:** 1 Center for Health and Bioresources, Molecular Diagnostics, AIT Austrian Institute of Technology GmbH, Giefinggasse, Vienna, Austria; 2 Bioinformatics Core Unit, Luxembourg Centre for Systems Biomedicine (LCSB), University of Luxembourg, Esch-sur-Alzette, Luxembourg; 3 Tytgat Institute for Liver and Intestinal Research, Amsterdam UMC, Location University of Amsterdam, Amsterdam, Netherlands; 4 Amsterdam Gastroenterology Endocrinology Metabolism (AGEM), Amsterdam, Netherlands; 5 Department of Gastroenterology and Hepatology, Amsterdam UMC, Location University of Amsterdam, Amsterdam, Netherlands; 6 Vatche and Tamar Manoukian Division of Digestive Diseases, University of California, Los Angeles (UCLA), Los Angeles, CA, United States; 7 Department of Medicine, David Geffen School of Medicine at UCLA, Los Angeles, CA, United States; 8 Goodman-Luskin Microbiome Center, University of California, Los Angeles (UCLA), Los Angeles, CA, United States; 9 G. Oppenheimer Center for Neurobiology of Stress and Resilience, UCLA, Los Angeles, CA, United States; 10 Institute of Clinical Molecular Biology, University of Kiel and University Hospital Schleswig-Holstein, Kiel, Germany; 11 Department Internal Medicine I, Kiel University, University Hospital Schleswig Holstein, Kiel, Germany

**Keywords:** DNA Methylation, fibrogenesis, multi-omics, neutrophil, severity, transcriptomics, ulcerative colitis

## Abstract

**Introduction:**

Ulcerative colitis (UC) is an idiopathic chronic inflammatory disease of the colon characterized by severe disease burden and multiple co-morbidities. Currently, the endoscopic Mayo score is considered the gold standard for assessing disease severity in UC. However, the molecular mechanisms underlying severity are still poorly understood. This study aimed to better understand the molecular alterations associated with severity in UC. To achieve this goal, gene expression and DNA methylation were measured in paired blood and colonic tissue samples from UC patients at different severity stages.

**Methods:**

Differential gene expression and methyl-ation analyses, as well as integrative multi-omics network analysis including both omics layers and tissues were performed. A hybrid framework combining prior knowledge and text-mining was deployed to contextualize the associations retrieved from the multi-omics network.

**Results:**

Our analyses suggested that mild UC was associated with molecular alterations affecting mainly the colon, while severe UC had systemic consequences. Moreover, the combination of the differentially expressed genes and methylated regions found in blood and colonic tissue allowed us to suggest potential associations between them and formulate hypothesis on novel mechanisms associated with different UC severity stages.

**Discussion:**

Among them, fibrogenesis, colonic epithelial cell death, and Tuft cell-related processes seemed to be associated with milder disease stages. In contrast, neutrophil-driven innate immune response and complex B cell and CD4 T cell interactions were suggested as potential mechanisms involved in severe UC. Finally, the findings of this study led to the formulation of a hypothesis suggesting that impaired PPARG anti-inflammatory regulation associated with colonic LCN2 activity might play a relevant role in UC severity.

## 1 Introduction

Ulcerative colitis (UC) is a chronic inflammatory disease characterized by relapsing and remitting mucosal inflammation (1). The pathogenesis of this disease is complex and still not completely understood. The currently accepted main hypothesis is that UC develops in people with a genetic predisposition exposed to certain environmental factors (2). Among them are exposures associated with gut microbiota, such as hygiene, antibiotic use or dietary factors, including the use of food additives (2–4). The worldwide incidence of UC has risen in the last decades (2, 3), reaching values ranging from 8.8 – 23.1 per 100,000 person-years in North America, 0.6 – 24.3 per 100,000 person-years in Europe and 7.3 to 17.4 in Oceania (2, 4, 5). An accurate assessment of the disease is essential, especially to evaluate the efficacy of the currently available treatments. The goal of any UC treatment is reaching and maintaining symptomatic and endoscopic remission. Nowadays, the most widely used clinical method to address disease severity and treatment efficacy is the Mayo score (MS) (6, 7). The original MS developed was based on stool frequency (SF), rectal bleeding (RB), the endoscopic appearance of the mucosa -recorded as an endoscopic score (ES)- and a Physician´s Global Assessment (PGA) (6). Both the European Medicines Agency (EMA) and the Food and Drug Administration (FDA) use a modified version of the Mayo score (mMS) which excludes PGA (6–9). Nevertheless, both agencies acknowledge the limitations of this index (7). In fact, the EMA endorses the endoscopic part of the MS (eMayo) as the best method to assess the efficacy of drugs against UC, even though it has not been fully validated yet (7, 9). Other endpoint assessments including histoendoscopic remission or comprehensive disease control, have been proposed, which combine different endpoints, e.g. histology, additional patient reported outcome parameters or simple inflammatory biomarkers (10). Thus, a better understanding of the molecular mechanisms underlying UC severity is needed. Given the molecular heterogeneity of patients, high-throughput omics technologies have emerged as powerful tools to advance the understanding of complexity in health and disease (10, 11). Although individual omics have revolutionized modern medicine, they are insufficient to fully capture the complex alterations underlying multifactorial diseases, like UC. Indeed, single-omics technologies focusing on only one type of molecule are largely correlative in nature and unable to capture the heterogeneity of the interactions occurring in different molecular events (12). However, combining different omics technologies enables the integration of a wider range of biological information; thereby improving our understanding of diseases and their molecular complexity (13). Robust and powerful bioinformatic tools, as well as advanced statistical approaches, need to be deployed to integrate heterogeneous and high-dimensional multi-omics data. Machine learning (ML) has proven to be very powerful in search of new clinically relevant patterns and reliable predictive markers of complex diseases (14). Therefore, the combination of multi-omics data with ML algorithms is a powerful approach to increase the current understanding of molecular mechanisms driving the severity of complex diseases, like UC. In fact, attempts to find novel molecular scores able to better assess inflammation in inflammatory bowel disease (IBD), combining transcriptomics and ML, have been carried out recently (15). However, they rely on pre-existing histological or clinical scoring methods, what limits their improvement in clinical applications. Therefore, the aim of this study was to combine ML and multi-omics data -consisting of transcriptomic and epigenomic measurements of paired blood and colonic tissue samples- to advance the current understanding of the molecular mechanisms driving disease severity, assessed with the eMayo score, in UC. Novel insights into pathophysiological mechanisms underlying disease severity will aid in developing more precise strategies for assessing risk and improving treatment approaches for UC.

## 2 Materials and methods

### 2.1 Study population

Blood and colonic tissue samples from UC patients, consisting of men and women in a wide age range (19–78 years) were included in this study. A total of 37 blood samples, 12 being paired measures at baseline and follow-up, from 31 patients constituted the first cohort. In addition, 60 colon biopsies, comprising 38 paired measures, from 41 patients were also included in this study. The harmonized population among blood and tissue comprised 35 samples, 8 of them being paired baseline and follow-up collected samples, from 31 patients. Only follow-up samples corresponded to samples collected after the administration of one of the following treatments: adalimumab (ADA), golimumab (GOLI), infliximab (IFX), tofacitinib (TOFA) and vedolizumab (VEDO). Three statistical groups were defined based on the eMayo score: (1) mild UC (N = 9), characterized by eMayo values of 0 or 1; (2) moderate UC (N = 16), defined by eMayo values of 2; and (3) severe UC (N = 10), represented by eMayo values of 3. A flow chart illustrating all the information explained above is available in supplementary materials (**Supplementary Figure 1**). Kruskal-Wallis test was deployed to compare all phenotypic traits available between groups. These were age, sex, body mass index (BMI), sustained remission, surgery, C-reactive protein (CRP), SF and RB. Pairwise comparisons were performed using Mann-Whitney test. All computed p-values were adjusted using the methodology developed by Benjamini and Hochberg (16).

### 2.2 Sample processing

Frozen gut biopsy tissue was mechanically homogenized in Lysis Matrix A tubes (MP Biomedicals) using a bead-based homogenizer, followed by combined RNA and DNA extraction with the Quick-DNA/RNA Miniprep Plus Kit (D7005, Zymo Research) according to the manufacturer’s instructions. For whole blood, total RNA and DNA were isolated separately each from 200 µL of frozen samples using the Quick-RNA Miniprep Plus Kit (R1057, Zymo Research) and Quick-DNA Miniprep Plus Kit (D4068, Zymo Research), respectively. Quality control of extracted nucleic acids included fluorometric quantification using a Qubit Flex Fluorometer (Thermo Fisher Scientific) and assessment of fragment size and integrity using a Fragment Analyzer (Agilent Technologies).

RNA-sequencing libraries were prepared according to the Illumina TruSeq^®^ stranded total RNA sequencing protocol (TruSeq^®^ Stranded Total RNA) and sequenced on an Illumina NovaSeq S4 (2 × 100 bp) aiming for a median depth of 30 million reads. DNA methylation analysis was performed with 500 ng of input DNA or, for low-yield samples, with the maximum available amount (≥250 ng). Bisulphite conversion was performed using EZ-96 DNA Methylation-Lightning Kit (D5033, Zymo Research), with successful conversion confirmed by qPCR. Finally, the samples were hybridized on Illumina Infinium Methylation EPIC BeadChip v1.0 microarrays, featuring more than 835,000 CpGs.

### 2.3 Statistical analysis of bulk-RNAseq data

Raw sequences were obtained in fastq format. An initial quality control was performed using FastQC (version v0.11.9) and multiQC tools (version 1.23) (17). Then, adapters were trimmed using Trim Galore (version 0.6.4), and the remaining reads were mapped against GRCh38 genome using STAR (version 2.7.6a) (18). Finally, the mapped.bam files were deduplicated, sorted and indexed for counting using picard tools (version 2.26.11) (19), samtools (version 1.7) (20) and htseq (version 0.11.3) (21) tools with default parameters, respectively. The mapped count matrices were further analyzed in R (version 4.2.2) (22) using Bioconductor packages (23). DESeq2 (version 1.44.0) (24) was utilized for data library-size normalization and regularized log transformation, whereafter limma (version 3.60.6) (25) was deployed for differential expression analysis. Sex and timepoint were included as fixed effects and patient as random effect to account for the timepoint effect. From the computed models, p-values and log2-transformed fold change (log_2_FC) values were extracted. The method developed by Benjamini & Hochberg was used for p-value adjustment (16) and the statistical significance was defined as adjusted p-value < 0.05. However, none of the transcripts analyzed met this criterion due to the limited sample size and the high dimensionality of the transcriptomic data. Given the exploratory nature of the present study, effect size was used as the selection metric. Thus, all the features displaying an absolute log_2_FC value >1 were considered differentially expressed genes (DEGs). To further investigate the immunological processes taking place at each severity stage, cell type proportions were predicted from bulk RNA-seq data using the R package granulator (version 1.12.0) (26) and different reference profiles for blood (27) and tissue (28, 29). In both cases, the deconvolution was carried out using the dtangle method (30) from granulator R package (26). In addition, non-parametric comparisons between groups were performed using the Kruskal-Wallis test to compute overall p-values and Mann-Whitney tests to compute pairwise p-values. The false discovery rate (FDR) was controlled following the Benjamini and Hochberg methodology (16). Then, a gene set enrichment analysis (GSEA) was performed to identify the pathways enriched in the DEGs discovered, using the biological process (BP) ontology from gene ontology (GO) database (31, 32). This analysis was computed using clusterProfiler R package (version 4.14.6) (33). Finally, a hierarchical clustering (HC) of the enriched terms based on their pairwise similarities, relying on Jaccard’s similarity coefficient, and Ward’s clustering method was created using enrichplot R package (version 1.24.4) (33).

### 2.4 Statistical analysis of DNA methylation data

Infinium MethylationEPIC v1.0 Kit was used to analyze the human methylation patterns of all the samples available for blood (N = 37) and tissue (N = 64). First, all the probes with detection p-values < 0.01 were excluded from the analysis to remove background noise arising from fault acquisitions. Then, raw data was normalized using the functional normalization procedure described in minfi (version 1.50.0) (34) R package. After normalization, DMRcate (version3.0.10) (35) R package was used to further pre-process the data as described in the following lines. All probes containing CpG sites closer than 2 bp from known single nucleotide polymorphisms (SNPs) and having a minimum minor allele frequency value lower than 0.05, as well as known cross-reactive probes with areas of the genome not at the site of interest and those hybridizing to sex chromosomes were removed. Once this was done, a differential methylation probe (DMP) analysis was conducted using limma R package (25), using the same design matrix used for RNA-sequencing. Then, differentially methylated regions (DMRs) were defined following DMRcate methodology using default parameters (35). From this analysis, minimum adjusted p-values from the CpGs constituting the DMRs and mean beta differences were obtained. An absolute mean beta difference value > 0.1 was used as a threshold to select significant DMRs, because all DMRs fulfilling this criterion also presented an adjusted p-value < 0.05. Finally, a gene ontology enrichment analysis was conducted using the R package missMethyl (version 1.40.3) (36). The HC of the enriched terms was created following the same methodology as that for bulk-RNAseq data (33).

### 2.5 Multi-omics statistical analysis

All paired blood and tissue (N = 35) samples were included in this analysis. Before conducting the multivariate analysis, Kruskal-Wallis and Mann-Whitney univariate tests were performed to assess differences in phenotypic features among groups. Then, a regularized canonical correlation (rCCR) model was computed to integrate the two omics layers (transcriptomics and epigenomics) and the two tissues (blood and colonic biopsy) using the R package mixOmics (version 6.28.0) (37). The transcriptomic input matrix comprised all the DEGs with absolute log2FC values > 1 in blood and colonic tissue, whereas additional steps were performed to obtain the matrix containing the epigenomic results used as input. First, the average M-values of all the CpGs collapsed in each DMR were computed, to assign a mean M-value to each DMR. Then, DMRs were sorted by absolute beta difference values and the 30% most differentially methylated DMRs, whose absolute difference in DNA methylation was > 0.1, were selected to ensure a comparable discovery depth across the two omics layers in the final model. Prior to modeling, both data matrices were mean-centered and scaled to the unit variance. Lambda values were optimized via 10-fold cross-validation. The scores of the first component of the rCCR were correlated with the log2-transformed CRP concentrations from the same patients, which is a well-known inflammation marker (38) and Pearson’s coefficient and p-values were computed.

### 2.6 Network and UC disease map analysis

Interactions between the features included in the rCCR were used to construct an undirected relevance network. A total of 615 interactions with an absolute associationvalue > 0.6 were kept in the network, to reduce background noise-derived complexity. Cytoscape (version 3.10.2) was used to visualize and customize the network. This threshold was found to be the best compromise between sparsity and density, in agreement with the results reported by the developers of the method (39). Additional networks consisting of the interactions of DEGs vs. DMRs in blood and DEGs vs. DMRs in tissue were also created (**Supplementary Figure 3**). The interaction terms from all reactions forming the network were designated as source and target, respectively. The similarity value between each pair of interacting elements, from now on *weight*, was also exported. This value was computed by calculating the sum of the correlations of the original variables and each latent component of the model (37). A detailed explanation on the computation of this value can be found in (39). In parallel, the contents of the latest version of UC disease map (UCdmap) (40) were transformed into a network consisting of 751 interactions. In addition, another network formed by 901,259 interactions and including at least one element from the data-driven network or UCdmap was created (TMnet) by querying a text mining database BioKB (41). TMnet contained multiple interactions between similar entities because of multiple evidence sentences for a single interaction, and different interaction types for the same molecule pair indicating positive or negative relationships. A positive sign (1) was given to reactions of the following types: *activates, increases, deubiquitinatesProtein, phosphorylatesProtein, glycosylatesProtein, deacetylatesProtein, demethylatesProtein, sumoylatesProtein, dehydroxylatesProtein, deribosylatesProtein, farnesylatesProtein*. A negative sign (-1) was given to reactions of the following types: *inhibits, decreases, ubiquitinatesProtein, dephosphorylatesProtein, deglycosylatesProtein, actelytatesProtein, methylatesProtein, desumoylatesProtein, hydroxylatesProtein, ribosylatesProtein, defarnesylatesProtein*. Although the weight was constructed to indicate uncertainty by how far it fell from 0, it may hide relevant opposing regulations, especially when multiple evidence sentences conflict. A complete text mining network (**Supplementary Table 5**) provided in the **Supplementary Materials** contains all individual interactions with their proposed sign and allows detailed lookup of compressed evidence. URLs in the table lead to sentences based on which an interaction has been constructed. This network was reduced to 88,361 interactions by computing a consensus direction for a given molecule pair by averaging the sign value across multiple interactions between the same molecule pair.

## 3 Results

### 3.1 Ulcerative Colitis severity cohort description

The current study comprised 37 blood samples from 31 patients (**Table 1**). In addition, 60 colonic biopsy samples were available from these 31 patients as well as from 10 additional patients (**Table 2**). For some of the patients – 6 in the case of blood samples and 19 in the case of colonic tissue - samples at two different timepoints -baseline and follow-up- had been collected. The statistical mixed-linear models accounted for this situation to avoid patient-derived bias in the analysis. All available samples were included when single blood and tissue analyses were performed, whereas in later integrative analyses a harmonized population consisting of 35 blood and colonic tissue paired samples corresponding to 31 patients was employed.

**Table 1:** Phenotypic trait comparison between severity groups for the population consisting of blood samples.

Variable	Mild (N = 9)	Moderate (N = 17)	Severe (N = 11)	Overall p-value	Overall adjusted p-value
Age (years)	34.0 [33.0;44.0]	32.0 [26.0;56.0]	39.0 [33.5;50.0]	0.553	0.553
Sex:				0.512	0.553
Female	8 (88.9%)	11 (64.7%)	8 (72.7%)	–	–
Male	1 (11.1%)	6 (35.3%)	3 (27.3%)	–	–
BMI (Kg/m2)	20.9 [19.2;21.9]	22.6 [21.3;28.7]	22.6 [19.7;25.1]	0.110	0.147
Sustained remission				0.077	0.123
No	3 (33.3%)	6 (35.3%)	6 (54.5%)	–	–
Yes	6 (66.7%)	4 (23.5%)	2 (18.2%)	–	–
Not Available	0 (0.00%)	7 (41.2%)	3 (27.3%)	–	–
CRP (mg/dL)	0.60 [0.55;1.55]	2.10 [0.80;9.70]	16.0 [6.35;27.8]	0.073	0.123
Stool frequency	0.00 [0.00;0.75]	2.00 [1.00;3.00]	3.00 [2.25;3.00]	0.017	0.068
Rectal bleeding	0.00 [0.00;0.75]	0.50 [0.00;1.75]	2.00 [1.50;3.00]	0.029	0.077
Timepoint				<0.001	0.008
Baseline	1 (11.1%)	15 (88.2%)	10 (90.9%)	–	–
Follow-up	8 (88.9%)	2 (11.8%)	1 (9.09%)	–	–

The number between brackets located below each severity group refers to the number of samples belonging to each group. Median and quartiles 1 and 3, separated by a semicolon, are reported for all numerical variables. Event number and relative percentages are displayed for all categorical variables. Overall non-adjusted p-values and false discovery rate adjusted overall p-values were also included.

**Table 2:** Phenotypic trait comparison between severity groups for the population consisting of colon biopsy samples.

	Mild (N = 21)	Moderate (N = 23)	Severe (N = 16)	Overall p-value	Overall adjusted p-value
Age (years)	38.0 [33.0;57.0]	35.0 [27.0;57.5]	43.5 [33.8;54.8]	0.744	0.759
Sex:				0.759	0.759
Female	15 (71.4%)	16 (69.6%)	13 (81.2%)		
Male	6 (28.6%)	7 (30.4%)	3 (18.8%)		
BMI (Kg/m2)	22.5 [20.3;24.1]	24.8 [21.3;28.6]	22.7 [20.2;24.7]	0.439	0.585
Sustained remission:				0.136	0.218
No	7 (33.3%)	8 (34.(%)	7 (43.8%)		
Yes	12 (57.1%)	6 (26.1%)	5 (31.2%)		
Not Available	2 (9.5%)	9 (39.1%)	4 (25.0%)		
CRP (mg/dL)	1.50 [0.60;4.30]	2.00 [0.60;4.20]	15.3 [5.20;35.6]	0.008	0.016
Stool frequency	0.00 [0.00;1.25]	2.00 [1.00;3.00]	3.00 [1.75;3.00]	0.003	0.008
Rectal bleeding	0.00 [0.00;1.00]	0.00 [0.00;2.00]	2.00 [2.00;3.00]	0.001	0.004
Timepoint				<0.001	0.004
Baseline	7 (33.3%)	19 (82.6%)	14 (87.5%)		
Follow-up	14 (66.7%)	4 (17.4%)	2 (12.5%)		

The number between brackets located below each severity group refers to the number of samples belonging to each group. Median and quartiles 1 and 3, separated by a semicolon, are reported for all numerical variables. Event number and relative percentages are displayed for all categorical variables. Overall non-adjusted p-values and false discovery rate adjusted overall p-values were also included.

Despite the slightly different number of patients, both blood and tissue populations were phenotypically comparable regarding their phenotypic characteristics. No differences were found in age (overall adjusted p-value = 0.553 and 0. 759 for blood and tissue, respectively), sex (overall adjusted p-value = 0.553 and 0.759 for blood and tissue, respectively), BMI (overall adjusted p-value = 0.147 and 0.585 for blood and tissue, respectively) and sustained remission (overall adjusted p-value = 0.123 and 0.585 for blood and tissue, respectively) among mild, moderate and severe UC. On the other hand, CRP was statistically significant in the cohort consisting of colonic tissue samples (overall adjusted p-value = 0.016) but not in that comprising blood samples (overall adjusted p-value = 0.123). This was also the case for stool frequency (overall adjusted p-value = 0.068 and 0.008 for blood and tissue, respectively) and rectal bleeding (overall adjusted p-value = 0.077 and 0.004 for blood and tissue, respectively). Nevertheless, the four variables displayed the same increasing trend with increasing severity; thereby reflecting the same clinical effect in both populations. Indeed, adjusted p-values, which were very close to the significance threshold of 0.05, were computed for these four phenotypic traits in the population consisting of blood samples. The lack of statistical significance was most likely caused by the smaller number of patients and not due to any additional effect that could confound the analysis. Finally, statistically significant differences were found regarding the timepoint at which blood and tissue samples were collected (overall adjusted p-value = 0.008 and 0.004 for blood and tissue, respectively). Most of the follow-up blood and colonic tissue samples, collected after treatment, corresponded to patients with mild UC (72% and 70%, respectively). All downstream statistical analyses performed accounted for the timepoint at which each sample was collected, in order to avoid any potential bias arising from the time of sample collection.

### 3.2 Mild ulcerative colitis involves localized alterations in the colon, whereas severe forms of the disease showed systemic effects.

**Figure 1:**
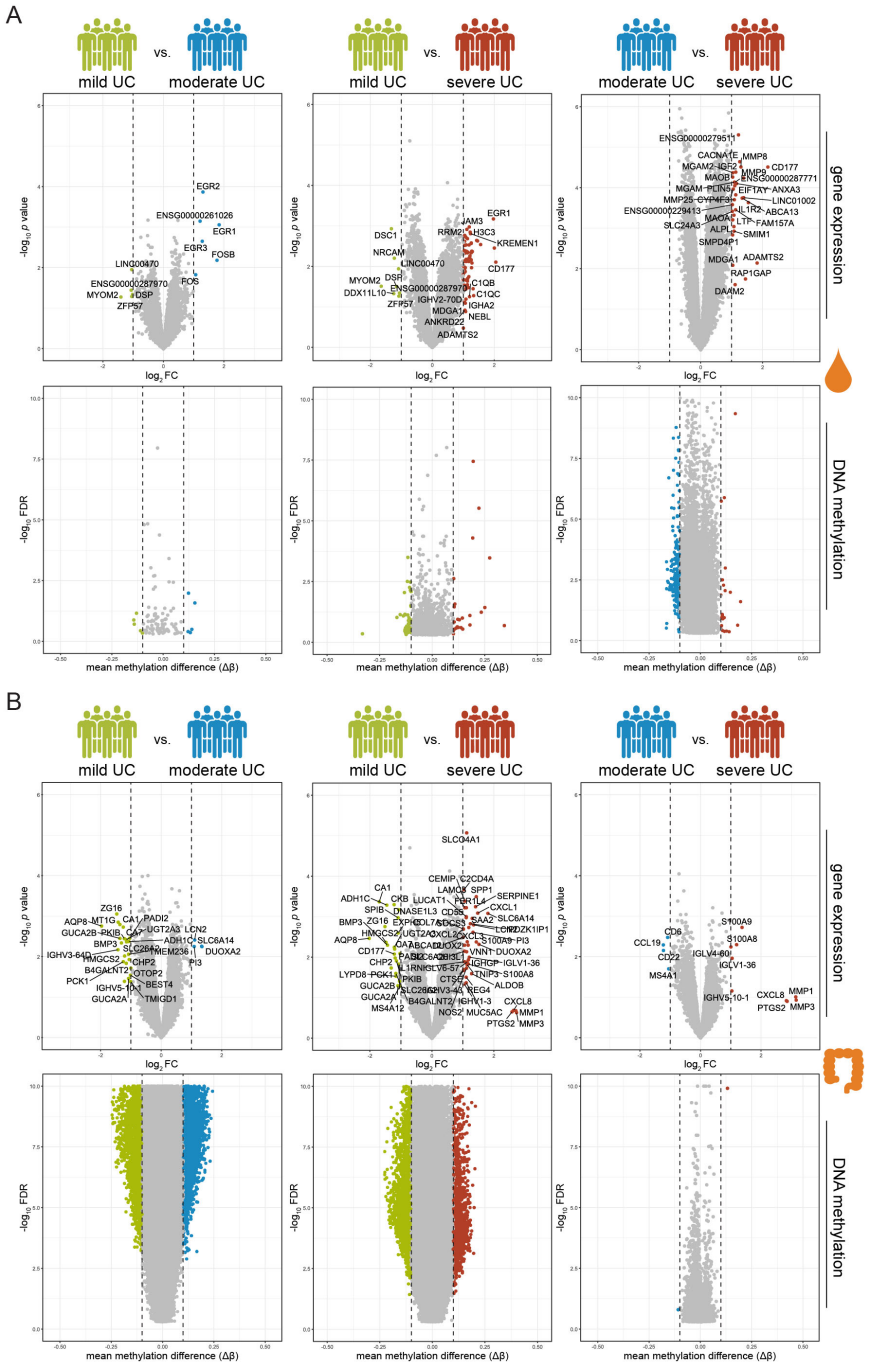
Differential gene expression and methylation analysis between ulcerative colitis severity groups. The color code used corresponded to each of the severity groups considered: (1) mild UC was highlighted in green, (2) moderate UC was colored in blue and (3) severe UC was marked in red. Each dot represents either a gene (**(A, B)**, top) or a methylated region (**(A, B)**, bottom). For the gene expression analysis (**(A, B)**, top) -log_10_-transformed p-values were displayed in the Y-axis, while log_2_FC values were included in the X-axis. For DNA methylation (**(A, B)**, bottom), the Y-axis showed minimum adjusted p-values from the CpGs constituting the DMRs, and the X-axis displayed mean beta differences. Pannel **(A)** corresponds to blood samples, whereas **(B)** displays the results obtained in tissue samples.

In blood, differential expression analyses identified 11, 78 and 31 DEGs showing an absolute log2FC value > 1 when comparing moderate UC (eMayo score = 2) with mild UC (eMayo score = 0 or 1), severe UC (eMayo score = 3) with mild UC, and severe UC with moderate UC, respectively (**Figure 1A**, top). Differential methylation analyses identified 10, 65, and 235 DMRs with an absolute beta difference value > 0.1 when comparing moderate UC to mild UC, severe UC to mild UC, and severe UC to moderate UC, respectively (**Figure 1A**, bottom). The same group comparisons were performed in tissue. In this case, 27 DEGs were found between moderate UC and mild UC groups, 66 between severe UC and mild UC groups and 13 between severe UC and moderate UC groups (**Figure 1B**, top). Although the total number of DEGs was similar between blood and tissue across all the comparisons, more DEGs were found between the severe UC and the remaining two groups in blood, whereas in tissue the largest number of DEGs was observed between mild UC and the other two groups. Finally, differences in DNA methylation in tissue were evaluated between severity groups (**Figure 1B**, bottom). The number of DMRs found was much larger compared to blood. In fact, 6,081 DMRs were found between moderate UC and mild UC groups, 3,876 between severe UC and mild UC groups and only 2 between severe UC and moderate UC groups. Taken together, these results indicated that both omics layers showed the same behavior in blood and tissue: the highest number of DEGs and DMRs between severe UC and the two milder severity stages in the blood, and between mild UC and the other two more severe groups in the tissue. In contrast, many transcriptomic and epigenomic changes were detected in colonic tissue from patients with mild UC and almost no differences were found between severe and moderate severity states.

**Figure 2:**
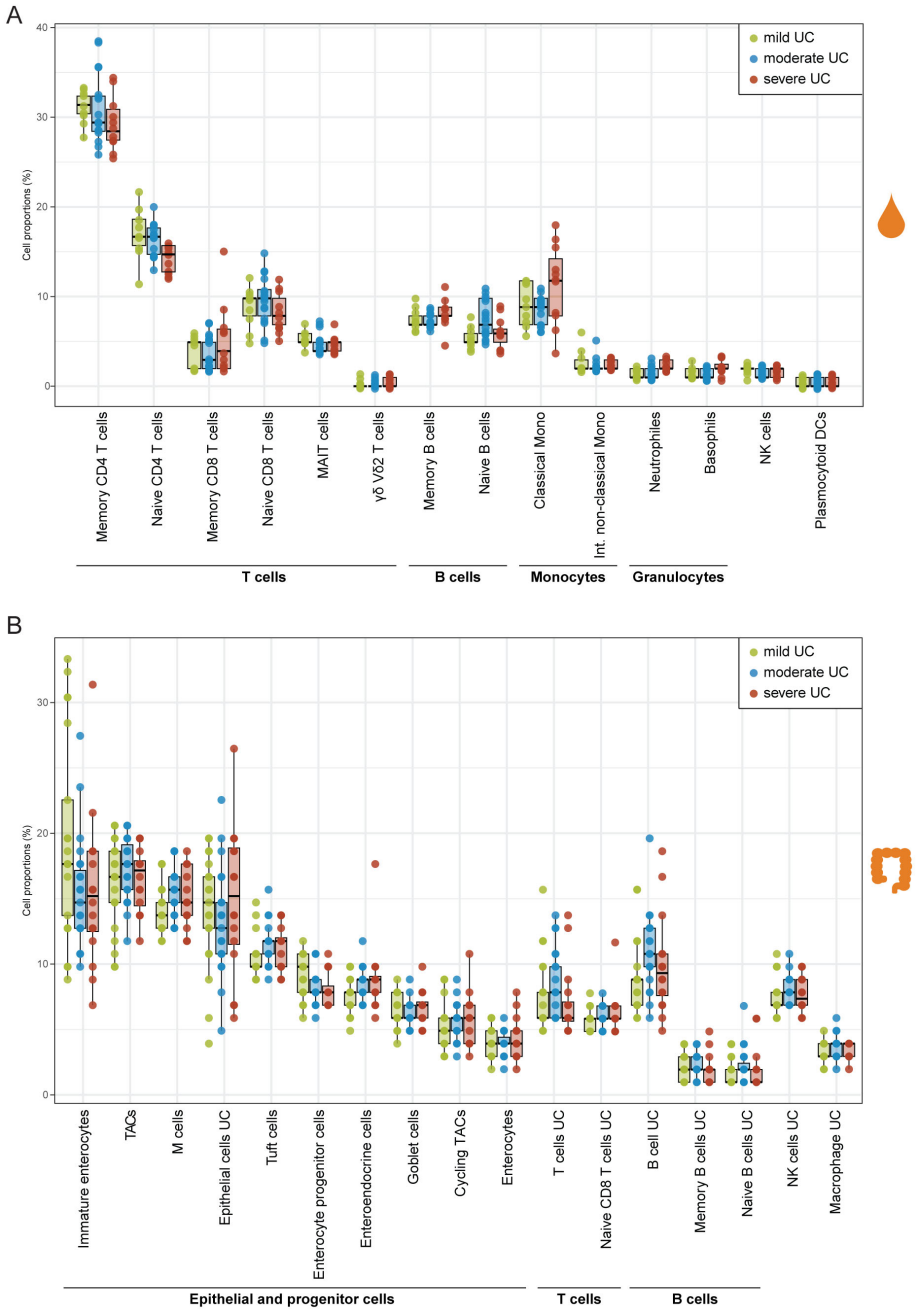
Cell type deconvolution. Computed cell proportions, in percentage, of several cell types in blood **(A)** and colon **(B)**. UC annotation accompanying several cell types deconvoluted in colon, indicates that these cell types were deconvoluted using a reference panel consisting of UC patients. Mucosal associated invariant T cells (MAIT cells), gamma delta Vd2 T cells (ϒ2 V δ2 T cells), monocytes (Mono), intermediate (Int), dendritic cells (DCs), natural killers (NK), transit amplifying cells (TACs), ulcerative colitis (UC).

To further investigate the immunological processes taking place on each matrix and at each severity stage, cell type fractions were predicted from transcriptomics data. In blood, a total of 14 immune cell type proportions were predicted (**Figure 2A**), and non-parametric comparisons between groups were computed (**Supplementary Table 1**). Among them, predicted cell proportions of both memory and naïve CD4 T cells decreased with increasing UC severity, although these effects were not statistically significant after false discovery rate adjustment (overall adjusted p-values = 0.384 and 0.088). No similar behavior was observed in either memory or naïve CD8 T cells, for which no noticeable differences were observed between severity states (overall adjusted p-values = 0.774 and 0.498, respectively). On the other hand, higher cell proportions of mucosal associated invariant T cells (MAIT) were found in mild UC. Nevertheless, this effect was not statistically significant (overall adjusted p-value = 0.380). The opposite effect was observed for ϒδ Vδ2 T cells, which were only detected in severe UC. B cells also showed some interesting effects. On the one hand, memory B cells were clearly increased in severe UC even if no statistically significant differences were observed (overall adjusted p-value = 0.380). On the other hand, naïve B cells displayed higher cell proportions in severe and especially in moderate states than in mild UC, despite these differences not being statistically significant (overall adjusted p-value = 0.144). In addition, several different immune cell types presented their maximum cell proportions in severe UC. These were: classical monocytes (overall adjusted p-value = 0.380), neutrophiles (overall adjusted p-value = 0.032) and basophils (overall adjusted p-value = 0.123).

Cell type deconvolution was also applied to tissue samples and 17 different cell type proportions were predicted (**Figure 2B**; **Supplementary Table 2**). Several epithelial and progenitor cells were identified. Among them, M cells (adjusted p-value = 0.013), Tuft cells (adjusted p-value = 0.009) and enteroendocrine cells (adjusted p-value = 0.051) showed increased cell proportion values in the between mild and moderate UC. These high cell proportion levels were also observed in severe UC for these cell types. On the other hand, a moderate decrease in cell proportions was found for goblet cells (overall adjusted p-value = 0.629), immature enterocytes (overall adjusted p-value = 0.182), and enterocyte progenitor cells (overall adjusted p-value = 0.182) between mild UC and more severe states - i.e. moderate and severe UC - of the disease. A similar effect was observed for epithelial cells (overall adjusted p-value = 0.492) too. For the remaining cell types included in the group of epithelial and progenitor cells, i.e., transit amplifying cells (TACs), cycling TACs and enterocytes, almost no differences were observed among the severity groups (overall adjusted p-value = 0.492, 0.518 and 0.918, respectively). In contrast to the deconvolution performed in blood, only a few T cell types were detected in colonic tissue. Unlike in blood, T cells and naïve CD8 T cells displayed their maximum proportion levels in moderate UC in tissue, even if these differences were not statistically significant (overall adjusted p-value = 0.182 and 0.492, respectively). The same trend was observed for B cells (overall adjusted p-value = 0.090), memory B cells (overall adjusted p-value = 0.387) and naïve B cells (overall adjusted p-value = 0.090). These differences were especially significant between moderate and mild UC (adjusted p-value = 0.015, 0.318 and 0.027, respectively). Additionally, NK showed higher cell proportions in severe and moderate UC than in mild UC (overall adjusted p-value = 0.143), being these differences especially significant between mild and moderate UC (adjusted p-value = 0.038). Finally, almost no differences were observed for macrophages (overall adjusted p-value = 0.518) between severity groups.

### 3.3 Differences in gene expression and DNA methylation associated with severity highlight distinct molecular pathways occurring in blood and colonic tissue.

**Figure 3:**
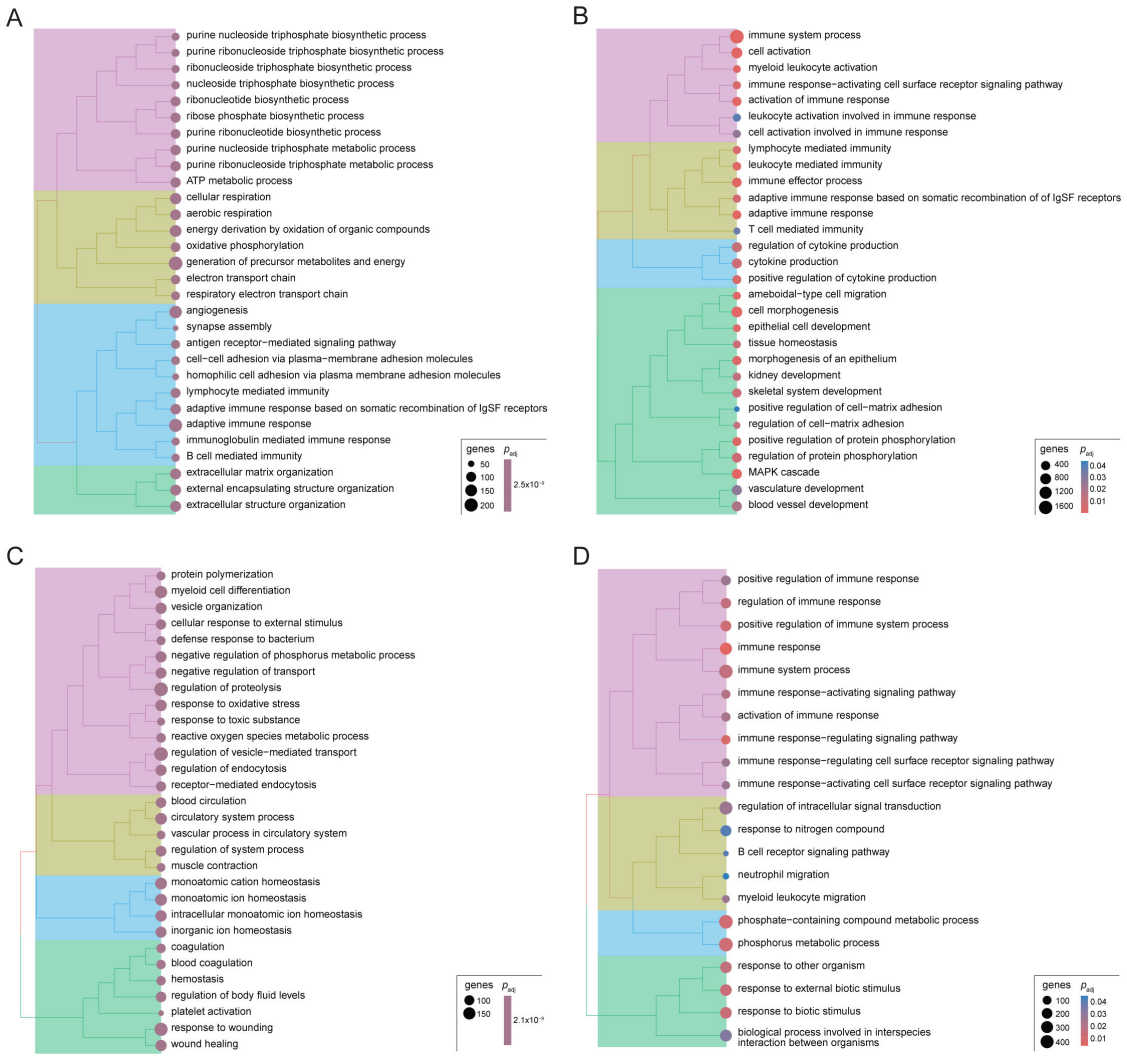
Biological processes enriched based on differentially expressed genes and differentially methylated regions. **(A, B)** Gene Ontology (GO) enriched biological processes between moderate and mild UC based on **(A)** DEGs and **(B)** DMRs in colonic tissue. **(C, D)** Gene Ontology (GO) enriched biological processes between severe and moderate UC based on **(C)** DEGs and **(D)** DMRs in blood. The size of the dots represents the number of genes or CpGs contributing to the pathway and the color to the adjusted p-value.

An enrichment analysis based on the differences in gene expression and DNA methylation observed at each severity stage was conducted to find biological pathways active at each of them (**Figure 3**). Regarding the comparison of mild and moderate UC, the differences observed in colonic tissue gene expression revealed the enrichment of biological processes associated with catabolism activation for ATP production, adaptative immune system and extracellular matrix (ECM) organization in colon (**Figure 3A**). On the other hand, when the changes in DNA methylation occurring in tissue were evaluated, most of the biological processes found were related to the activation of humoral and cell-mediated adaptative immune response and cytokine production (**Figure 3B**). Additionally, several biological processes involved in epithelial tissue homeostasis, epithelial cell morphogenesis, cell-matrix adhesion and angiogenesis were also highlighted, as well as the MAPK pathway (**Figure 3B**). Then, biological processes enriched in severe UC and based on transcriptomics and epigenomics changes in blood, were inspected. The DEGs found in blood at severe UC stage were associated with innate responsive mechanisms against biological stress (proteolysis, reactive oxygen species production or endocytosis), cell circulation and recruitment, ion homeostasis, hemostasis and wound healing mechanisms (**Figure 3C**). Finally, the enrichment analysis based on DNA methylation changes observed in blood highlighted biological processes playing a role in innate immune response activation (especially neutrophil mediated response), B cell stimulation, phosphate-containing molecules metabolism and response to biotic stimulus in severe UC (**Figure 3D**).

### 3.4 Combined transcriptomic and epigenomic profiles in blood and colonic tissue captured molecular alterations associated with different UC severity stages.

To integrate the two omics layers (transcriptomics and epigenomics) measured in blood and colonic tissue, only paired blood and tissue measures (N = 35) were selected from the available samples. Prior to multivariate analysis, the phenotypic differences between severity groups were analyzed (**Table 3**). No statistically significant differences for age (overall adjusted p-value = 0.598), sex (overall adjusted p-value = 0.598), BMI (overall adjusted p-value = 0.128), nor for sustained remission (overall adjusted p-value = 0.128) were observed. However, all the clinical features associated with severity of UC showed stronger differences between study groups. These were: CRP (overall adjusted p-value = 0.090), stool frequency (overall adjusted p-value = 0.090), and rectal bleeding (overall adjusted p-value = 0.090).

**Table 3:** Phenotypic trait comparison between severity groups for the harmonized blood and colon biopsy paired population.

Variable	Mild (N = 9)	Moderate (N = 16)	Severe (N = 10)	Overall p-value	Overall adjusted p-value
Age (years)	34.0 [33.0;44.0]	30.0 [25.8;57.8]	39.5 [33.2;50.0]	0.529	0.598
Sex:				0.598	0.598
Female	8 (88.9%)	11 (68.8%)	8 (80.0%)		
Male	1 (11.1%)	5 (31.2%)	2 (20.0%)		
BMI (Kg/m2)	20.9 [19.2;21.9]	24.8 [21.5;28.7]	21.4 [19.7;23.9]	0.061	0.128
CRP (mg/dL)	0.60 [0.30;1.50]	2.40 [0.78;10.5]	17.6 [3.42;30.1]	0.03	0.0900
Stool frequency	0.00 [0.00;0.75]	2.00 [1.00;3.00]	3.00 [2.25;3.00]	0.017	0.0900
Rectal bleeding	0.00 [0.00;0.75]	0.50 [0.00;1.75]	2.00 [1.50;3.00]	0.029	0.0900

The number between brackets located below each severity group refers to the number of samples belonging to each group. Median and quartiles 1 and 3, separated by a semicolon, are reported for all numerical variables. Event number and relative percentages are displayed for all categorical variables. Overall non-adjusted p-values and false discovery rate overall adjusted p-values were also included.

**Figure 4:**
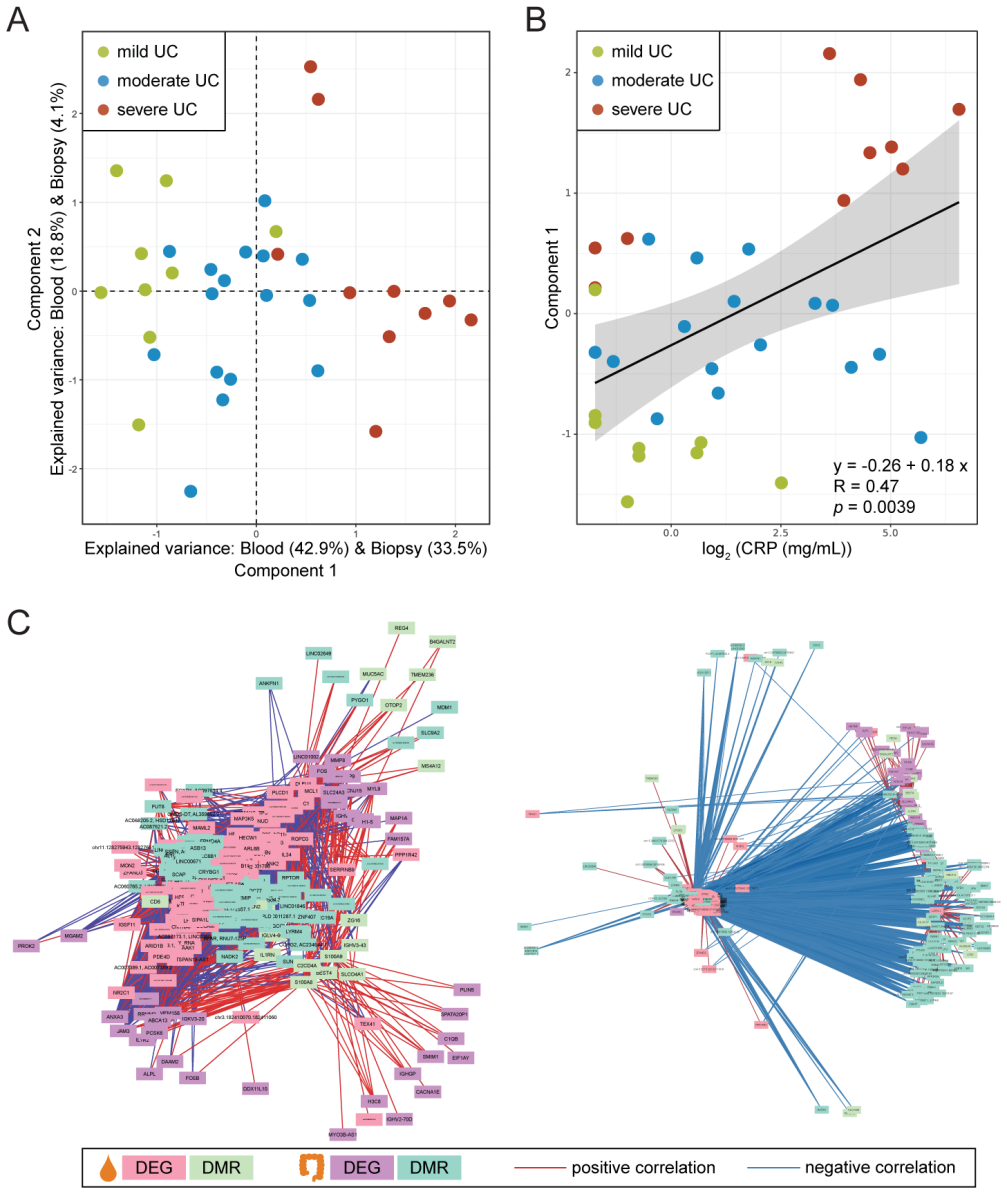
Regularized canonical correlation integrating gene expression and DNA methylation from blood and tissue. **(A)** The distribution of patient’s scores in the two-dimensional space delimited by the first two components of the rCCR. **(B)** Scatter plot of the correlation of component 1 scores (Y axis) vs. log2-transformed CRP concentrations (X axis). The equation of the linear model, as well as Pearson’s coefficient (R) and the p-value were indicated on the bottom-right part. **(C)** Two different layouts of the network displaying the interactions underlying the canonical correlation model. The network consisted of two different types of interactions: (1) DEG in blood vs. DEG in tissue, (2) DEG in blood vs. DMR in tissue, (3) DMR in blood vs. DEG in tissue and (4) DMR in blood vs. DMR in tissue. Nodes were highlighted as follows: (1) DEG in blood were colored in pink, (2) DEG in tissue were colored in purple, (3) DMR were colored in light green and (4) DMR in tissue were colored in dark green. Red edges represented positive correlations between the two nodes connected, whereas blue edges corresponded to negative correlations between them. Differentially expressed gene (DEG), differentially methylated region (DMR).

The most relevant DEGs and DMRs showing the highest differences between severity groups were selected. Then, existing overlaps between blood and tissue were evaluated, to find common DEGs and DMRs in both biological matrices (**Supplementary Figure 2**). Nevertheless, only 2 of the top DEGs (1.2%) and 3 of the top DMRs (0.6%) highlighted from the differential gene expression and DNA methylation analyses were found both in circulation and in colonic tissue. Analysis of such a complex phenomenon in UC severity was done by building a rCCR model using the top DEGs and DMRs found in blood and tissue. This approach enabled the calculation of a single score for each patient, reflecting the combined effects of their transcriptomic and epigenomic profiles. The scores were then visualized in a reduced two-dimensional space delimited by the first two components of the rCCR (**Figure 4A**). The first principal component, which was able to capture almost half of the total variance, led to a clear separation between patients with mild, moderate, and severe UC. Patients with mild UC had low values of this component, whereas for patients with severe UC high values of the first component were observed. Finally, patients with moderate UC presented intermediate values of component 1. To further verify that the first component of the rCCR was truly capturing a biological effect and not a statistical artifact, the scores of this component were correlated with the CRP levels from the patients (**Figure 4B**). CRP is a well-known indicator of inflammation, and therefore of severity, in many diseases (37). A significant correlation (p-value = 0.0039) was observed between patients’ CRP levels and the first component of the model computed. Moreover, this analysis showed a coherence between CRP and component 1 values. For most cases, higher and lower CRP values corresponded to higher and lower values of component 1, respectively.

### 3.5 Associations between DEGs and DMRs in blood and colonic tissue identified potential novel mechanisms related with UC severity.

The last part of the study focused on further investigating the biological role of the DEGs and DMRs included in the model described in the previous section. For this purpose, the interactions between DEGs and DMRs from blood and tissue were displayed in a partially connected network (**Figure 4C**). This network consisted of four types of interactions: (1) DEGs found in blood with DEGs found in tissue, (2) DEGs found in blood with DMRs found in tissue, (3) DMRs found in blood with DMRs found in tissue and (4) DMRs found in blood with DEGs found in tissue. On the one hand, strong positive associations between DEGs from blood and tissue could be observed in the network’s layout displayed on the left part from **Figure 4C**. In fact, all the interactions between DEGs in blood and DEGs in tissue corresponded to positive correlations. On the other hand, negative associations between DNA methylation in blood and gene expression in blood and tissue could be clearly observed in the alternative layout (**Figure 4C**, right). Moreover, most of the tissue DEG and DMR were also separated from each other. This spatial feature distribution enhanced the trustworthiness of the network, since it was able to capture the known general inhibitory effect of DNA methylation on gene expression (42), even if the interactions of DNA methylation and gene expression within blood and tissue were not directly included in it. This inhibitory effect was further assessed when additional networks consisting of DEGs and DMRs interactions for the same biological matrix were created (**Supplementary Figure 3**).

**Figure 5:**
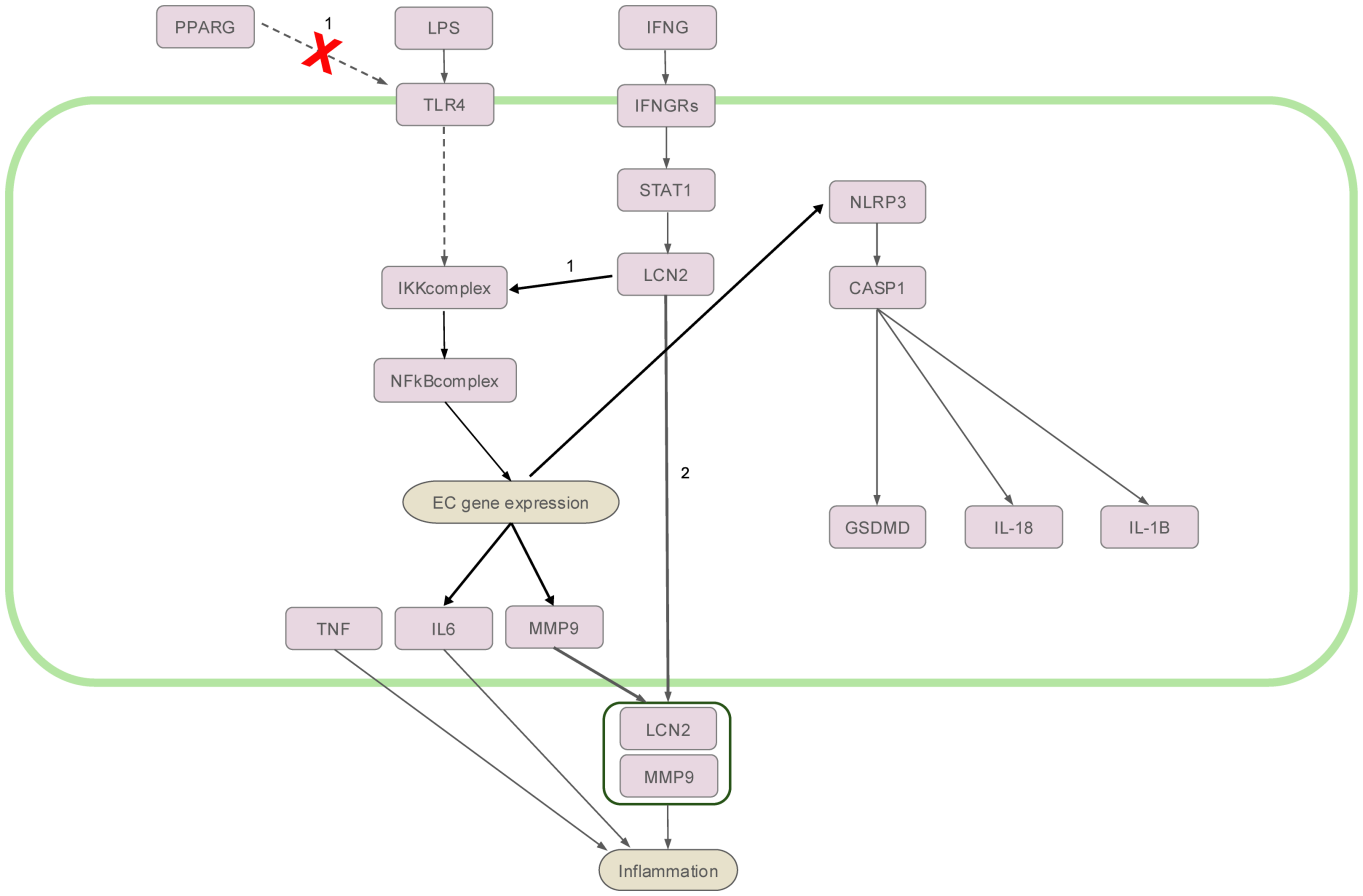
Diagram displaying the two molecular mechanisms suggested. The associations among the candidates involved in each mechanism are highlighted either with 1 or 2. Purple rectangles represent proteins, while yellow ones represent biological processes. Yellow circles correspond to molecules different from proteins. The connections between the elements involved in each mechanism were either direct (continuous arrows) or indirect (discontinuous arrows) A green rectangle was used to represent the colon, while the part outside of it corresponded to peripheral blood.

To find novel mechanisms involved in UC severity, the DEGs and DMRs acting as nodes in the data-driven network were overlapped with the proteins included in the UCdmap, which is a manually curated and literature supported systems biology diagram describing disease mechanisms (40). Because the interactions constituting the data-driven network were based on the association between the two nodes (DEGs or DMRs from blood or colonic tissue), they did not necessarily indicate a direct connection between the two proteins but a positive or negative relationship among them. Hence, all shortest paths in the UCdmap involving proteins highlighted in the data-driven network were analyzed. This analysis reported 92 interactions in the UCdmap including the proteins from the data-driven network. Potential intermediates connecting the source and target proteins from the 92 interactions found were also evaluated. This was performed by looking for common targets, in UCdmap, for each pair of molecules involved in each of the 92 interactions found (**Supplementary Figure 4**). In addition, TMnet consisting of more than 900,000 literature-supported protein interactions, was used to validate novel interactions involving intermediate proteins, as well as their directionality. This analysis revealed 467 protein pairs from the data-driven network that had at least one common target included in the UCdMap. Furthermore, for 231 of them, their correlation sign agreed with the consensus direction computed for TMnet interactions. Finally, for 164 protein pairs, at least one member of the pair was not included in the UCdmap, thereby suggesting potential novel events associated with UC severity. From them, a total of 32 interactions were prioritized based on the importance of their interacting terms (DEGs or DMRs) in the canonical correlation model (**Supplementary Table 3**). These interactions came to 2 potential molecular mechanisms associated with UC severity and involving cross-tissue and cross-omics LCN2 and PPARG associations, given that 13 out of the 32 interactions involved these two markers (**Figure 5**; **Supplementary Table 3**). The first mechanism suggested a potential negative association between PPARG DNA methylation levels in peripheral blood mononuclear cells (PBMCs) and the colonic expression of LCN2 and TLR4. In contrast, the second one suggested a potential positive association between the expression of LCN2 in colonic tissue and MMP9 in peripheral blood, being both of them negatively associated with PPARG DNA methylation levels in PBMCs. These hypothesis-driving interactions were reproduced in an independent cohort (15) consisting of paired blood and biopsy samples from patients with UC (**Supplementary File 2**).

## 4 Discussion

This study aimed to expand the current knowledge of the molecular complexity underlying UC severity by identifying novel potential associations between DEGs and DMRs measured in paired blood and colonic tissue samples from UC patients across different stages of disease severity. The biological plausibility of the data-driven associations was assessed in the context of the extensive existing literature using a novel framework that integrates text-mining with publicly available UCdmap resource. This analysis enabled the formulation of strong hypotheses suggesting potential novel molecular mechanisms associated with UC severity. Our investigation into differential gene expression and methylation associated with the different severity stages showed that mild and moderate UC shared more similar transcriptomic and epigenomic profile in blood, with the largest number of alterations occurring at a severe stage of UC. These results suggest that milder UC stages are mostly confined to colon and trigger local molecular alterations, whereas more severe forms of the disease have systemic consequences.

The results obtained in this study pointed to three major biological events that migth be occuring at milder stages of the disease: (1) promotion of fibrogenesis caused by ECM accumulation, (2) epithelial barrier impairment due to increased cell death and (3) tuft cell decrease. Recent research increasingly highlighted the key role of fibrogenesis in the UC severity (43–45). This is a complex process characterized by an excessive accumulation of ECM –comprising a heterogeneous cell population of mesenchymal cells, such as fibroblasts, myofibroblasts and smoothed muscle cells– within and around damaged tissue (46, 47). In our study, several enriched biological processes related to ECM organization and cell–matrix adhesion were identified exclusively in colonic tissue when the gene expression and DNA methylation profiles of patients with mild were compared to those of patients with moderate UC. Since UC progression is not a linear process (1, 48), and some patients included in this study underwent different treatments, our results suggest the presence of fibrotic activity even after the therapeutic suppression of inflammation. This hypothesis aligns with recent studies indicating that fibrosis may be initiated in the absence of a strong inflammatory response but progresses in parallel with inflammation (44, 45, 47). Moreover, while inflammation is required to initiate fibrogenesis, the reduction of inflammation due to therapeutic approaches does not prevent fibrosis development (47). In addition, B cells might also play a relevant role in fibrogenesis, since they accumulate in inflamed intestine and in the lamina propria of UC patients (49–51). A recent study (52) suggested that the space occupied by B cell accumulation reduces the interactions between stromal and epithelial cells in colon, thereby blocking tissue healing. Our results showed an early and significant increase of B cells in colonic tissue in moderate UC. Moreover, unlike all other immune cell types that increase at the moderate stage in colon, B cells did not maintain this increase at the severe stage. This result suggests a transient increase in B cells infiltration in colon, which aligns with previous studies (44, 45) suggesting that immune mechanisms are necessary to initiate fibrogenesis, but its progression can occur independently of maintained inflammation. Regarding colonic epithelial barrier integrity, the alterations in DNA methylation observed between mild and moderate UC were associated with tissue morphogenesis and homeostasis, but none of them were specifically linked to tissue healing, which was only enriched in severe UC. In contrast, several pathways associated with cytokine production were significantly enriched based on differences in gene expression between mild and moderate UC. In addition, cell-type deconvolution results showed a decrease in colonic epithelial, immature enterocytes and enterocyte progenitor cells -which are key contributors to tissue healing due to their high regenerative potential- between mild and moderate UC (53). All these findings might indicate an increased epithelial cell death rate in moderate UC, which is frequently observed in UC patients (54, 55). The last major effect suggested by our results was the decrease of tuft cells in mild UC, compared to moderate and severe UC. Tuft cells have been recently highlighted as a potential key actor in UC and colon mucosa inflammation, even if its role has not been clarified yet (56). In contrast to our findings, the limited literature available on this topic has reported decreased values of tuft cells in IBD (57, 58). However, the first study (57) only included healthy control and UC groups and did not evaluate relative differences between severity-based groups, and the second one (58) only included patients with Crohn’s disease (CD), which -unlike UC- is characterized by a type I immune response. Indeed, tuft cells are the main productors of IL-25, which promotes type 2 immune response (56, 57). Nonetheless, a recent study reported increased mucosal tuft cell density in colon biopsies from patients with diarrhea-predominant irritable bowel syndrome (IBS), while their density was not increased in patients with any of the non-diarrheic subtypes of IBS (59). This is a relevant finding, since diarrhea is a sign of active UC (8, 9).

As mentioned above, our results suggested an expansion of UC beyond colon mucosa in a severe stage of the disease. In fact, several pathways associated with the activation of immune response against gut microbiota were found to be significantly enriched in blood at severe UC. This expansion has been discussed in diverse studies (43, 60–62) and is known to lead to systemic inflammation in the severe stage of the disease (62, 63). Whereas the immune processes observed in the colon at milder stages of UC were primarily driven by components of the adaptive immune response, such as B and T cells, the enriched biological processes identified in blood at more severe stages of the disease were predominantly associated with innate immune responses. Changes in gene expression were highly associated with characteristic processes of innate immune response, like myeloid cells differentiation, endocytosis, proteolysis, oxidative stress, and reactive oxygen species (ROS) production (62, 64). Moreover, DNA methylation changes also highlighted natural processes of innate immune response, such as myeloid leukocyte and neutrophil migration. Nonetheless, DNA methylation also highlighted mechanisms known to be part of the adaptive immune system like B cell receptor (BCR) signaling pathway. In fact, the results obtained in this study pointed towards two major processes dominating the immune response occurring at severe UC: (1) neutrophil activation and (2) B and T cell interactions. On the one hand, our results showed a significant increase of circulating neutrophils in severe UC. This aligns with several studies reporting increased circulating neutrophil concentrations in UC to be associated with disease activity and severity (65–67). Moreover, the recruitment and activation of neutrophils contribute to disease pathology through the release of harmful agents such as cytotoxic enzymes, ROS, and diverse proteases, along with massive transepithelial migration, all of which result in extensive mucosal injury (62, 67). On the other hand, BCR recognizes antigens in response to viral and bacterial infections, and this is the first step for enabling the differentiation of naïve B cells towards memory B cells mediated by helper T cells (also known as CD4 T cells) (68, 69). Our results might have reproduced this effect, since they showed an increase of circulating naïve B cells at the moderate stage of UC, which subsequently led to a rise of memory B cells at the severe stage. However, our results also showed a decrease in nave and memory CD4 T cells with increasing severity. A recent study focusing on UC severity reported the same trend (70). A possible hypothesis for this effect may rely on the circulating CD4 T cell subpopulations and their differential roles in UC pathogenesis. Indeed, a recent study found altered functional subpopulations of circulating CD4 T cells in patients with UC and linked these alterations with increased B cell proportions, especially with memory B cells (51). Our results might reflect changing CD4 T cell subpopulation ratios with increasing severity. The decrease in CD4 T cells between mild and moderate UC was not very pronounced, especially in naïve CD4 T cells and coincided with a significant increase of naïve B cells activated in moderate UC. This effect may be due to a higher proportion of follicular helper CD4 T cells (Tfh) relative to follicular regulatory CD4 T cells (Tfr) in moderate UC. Tfh cells promote B cell activation, whereas Tfr cells inhibit it, suggesting that the altered Tfh/Tfr balance—rather than an overall increase in CD4 T cells—drives this effect (51, 71). This relationship may be reversed at the severe stage, possibly due to a greater decrease in Tfh cells compared to Tfr cells, leading to a reduction in naïve B cell levels and a reliance on memory B cells for the immune response. However, this remains a hypothesis and requires validation through additional experiments, such as single-cell sequencing or other high-resolution approaches.

Thus far, several biological processes that appear to be associated with UC severity have been discussed. However, within the scope of this hypothesis-generating study, we also aimed to explore and identify novel potential associations between gene expression and DNA methylation signatures from paired blood and colonic tissue samples, that could provide insights into novel molecular mechanisms driving the severity of UC. Indeed, the limited overlap of genes that were differentially expressed or methylated across both blood and colonic tissue, as well as the small number of genes showing concurrent alterations in DNA methylation and gene expression within the same tissue, underlines the multifactorial and complex nature of UC severity. Our results showed that different biological processes were associated with different severity stages in blood and colonic tissue. However, this fact does not necessarily imply that the markers identified through gene expression and DNA methylation analyses were not involved in the same broader process, such as innate and adaptative immunity, tissue homeostasis or other severity-related biological processes. In fact, under a pathogenic stimulus, a dynamic process like DNA methylation is likely to modulate and control several DEGs to fine-tune processes such as immune response (72).

To explore cross-tissue multi-omics associations, we used a novel framework that first identified multi-omics interactions between tissues and subsequently explored their possible biological relevance by contextualizing them throughout the extensive literature available for UC severity. This analysis came to 2 potential molecular mechanisms involved in the severity of UC. On the one hand, the data-driven network showed strong negative associations between DNA methylation levels of PPARG derived from PBMCs and colonic expression levels of LCN2. Although no direct interactions between these two proteins had been described, our novel approach was able to find diverse intermediate proteins connecting both, like TLR4. The results of this study revealed low methylation levels of PPARG in PBMCs- suggesting an increased expression of this gene in them- coexisting with increased colonic expression of LCN2 and enhanced TLR4 signaling, as a novel potential molecular mechanism contributing to the severity of UC. Moreover, the trend of this finding was reproduced in an independent dataset consisting of gene expression levels from paired blood and colonic biopsy samples, as previously stated. While a mechanism involving all three candidates has not been previously described, all the markers involved in it are known to play a relevant role in the severity of UC (54, 73–76). On the one hand, LCN2 expression in colon has been reported to increase following TLR4-mediated microbial stimulation (62). Furthermore, high expression of LCN2 has been associated with pro-inflammatory processes in colonic epithelial cells (54). In addition, several additional intermediate candidates identified between PPARG methylation in PBMCs and LCN2 expression in colonic tissue, including NOS2, TNF-α, IL6 and IL1B, correspond to pro-inflammatory mediators that have been reported to be upregulated following LCN2-induced activation of NF-κB (54, 77, 78). This observation further supports the existence of a common inflammatory process linking PPARG epigenetic alterations in PBMCs with local mucosal inflammation. While diverse studies have investigated the interaction of TLR4 and colonic PPARG in UC (74–76), our findings suggest, for the first time, a potential negative association with PPARG methylation in PBMCs. Indeed, PPARG is expressed in PBMCs among other tissues, where it plays an anti-inflammatory role by downregulating the production of cytokines, such as TNF-α and NF-κB (79). Thus, the role of PPARG in the hypothesized mechanism remains puzzling, since higher expression of this gene would be expected to be associated with lower inflammation. Nonetheless, its association with colonic LCN2 expression -which increases with disease severity- might suggest that PPARG hypomethylation could be a compensatory response triggered to control inflammation. Indeed, a recent study evaluated the effect of PAR5359, a PPARG agonist, on enhancing bacterial clearance capacity of PBMCs from patients with UC and CD (80). While PAR5359 significantly increased the bacterial clearance of PBMCs from CD patients, no such effect was observed in PBMCs derived from UC patients (80). These findings aligned with our results, which suggested no beneficial effects of PBMCs-derived PPARG in inflammation driving UC severity. Reduced bacterial clearance activity from PBMCs might lead to an enhanced TLR4 expression and subsequent LCN2 cascade activation. Collectively, these observations might contribute to formulating further hypotheses about a potential impairment of PPARG-driven anti-inflammatory response specific to UC.

Another intermediate marker between PPARG and LCN2 found in the analysis was MMP9. In this case, lower PPARG DNA methylation levels in PBMCs were accompanied by increased expression of LCN2 and MMP9 in colonic tissue and circulation, respectively. The gene expression levels of the three markers, assuming an inhibitory effect of DNA methylation in PPARG, increased with disease severity. This trend was observed again using the previously mentioned independent cohort (15). This finding suggested the involvement of neutrophils, given that an increased proportion of them was one of the main findings at severe UC. Indeed, neutrophils secrete tertiary granules that contain MMP9 (73), which can form a covalent complex with LCN2, whose levels in circulation are associated with disease severity in UC (49, 73). The role of PPARG expression in PBMCs as a mechanism to decrease neutrophil recruitment and promote neutrophil apoptosis has been previously described (81). However, our results revealed an increased neutrophil proportion in severe UC. In line with the previously mentioned study, which reported that the PPARG agonist PAR5359 was unable to restore microbe clearance in PBMCs extracted from patients with UC (80), the large proportion of neutrophils observed in severe UC despite PPARG hypomethylation might indicate an impairment of PPARG-mediated regulatory pathways in these cells. Such dysfunction could compromise neutrophil population control, which may favor the formation of the LCN2-MMP9 complex, with LCN2 originating from colonic tissue and MMP9 from circulating neutrophils.Some limitations must be acknowledged in this study. On the one hand, the limited sample size compromised the statistical power of differential expression and methylation analyses, and this could introduce bias in the selection of the most relevant DEGs and DMRs. Nonetheless, the main goal of this study was to evaluate potential associations between gene expression and DNA methylation signatures measured in colonic tissue and blood, and to contextualize their biological plausibility through a novel approach involving the use of text-mining in combination with publicly available UC disease maps. Indeed, the markers selected enabled the evaluation of more than 700 candidate interactions. In addition, the results obtained from all the comparisons between groups performed in this study were relative changes within the disease due to the lack of a healthy control group. In addition, our cohort consisted of pre-treatment and post-treatment collected baseline and follow-up samples. On the one hand, all analyses accounted for the biological variability arising from the inclusion of both baseline and follow-up samples for some patients within the cohort. On the other hand, the potential effects of the five different treatments administered could not be considered, since incorporating such a granular variable into the models would have dramatically reduced their stability, given the limited sample size of the study cohort. Nevertheless, considering the diversity of treatments, no treatment-specific effects attributable to single drugs were expected. Almost all the samples collected after treatment belonged to the mild UC group, suggesting that mild severity stage was induced by the treatment in several cases. However, this represented a strength rather than a limitation since, as stated above, UC progression is a heterogeneous process characterized by having relapsing and remitting phases (1, 48). Therefore, the inclusion of both treatment-free and -induced mild UC samples reflected more accurately the real progression of the disease. Indeed, the heterogeneity of our cohort reflects the variability encountered in real-world clinical practice, supporting the real-life relevance of our findings. Furthermore, this study has several additional strengths. First, the availability of transcriptomic and genomic data from paired blood and colonic tissue samples allowed us to evaluate not only tissue-specific processes but also cross-tissue molecular mechanisms. Moreover, we developed a novel framework capable of contextualizing data-driven multi-omics and multi-tissue associations with the existing prior-knowledge in the field, which allowed us to formulate strong hypothesis that should be further validated in future studies. Indeed, public accessibility of UCdmap is a valuable resource that will support researchers in future studies.

In conclusion, the results obtained in this study allowed us to formulate mechanistical hypothesis about molecular alterations in blood and colonic tissue that may contribute to severity in UC. As a hypothesis-generating study, it may provide a valuable starting point for future investigations that could attempt to validate the proposed mechanisms.

## Data Availability

The datasets used and/or analyzed during the current study are available from the corresponding author on reasonable request. The raw data supporting the conclusions of this article will be made available by the authors, without undue reservation.

## Glossary

ADA: Adalimumab
ALOX15: Arachidonate 15-Lipoxygenase
BCR: B Cell Receptor
BMI: Body Mass Index
BP: Biological Process
CD: Crohn’s Disease
CRP: C-reactive protein
DEG: Differentially Expressed Genes
DMP: Differentially Methylated Probe
DMR: Differentially Methylated Region
ECM: Extracellular Matrix
EGR1: Early Growth Response 1
EMA: European Medicines Agency
eMayo: Endoscopic Mayo Score
ES: Endoscopic Score
FC: Fold Change
FDA: Food and Drug Administration
FDR: False Discovery Rate
GO: Gene Ontology
GOLI: Golimumab
GSEA: Gene Set Enrichment Analysis
HC: Hierarchical Clustering
IBD: Inflammatory Bowel Disease
IBS: Irritable Bowel Syndrome
IL: Interleukin
IFX: Infliximab
LCN2: Lipocalin-2
M: Methylated
MAIT: Mucosal Associated Invariant T cells
MAPK: Mitogen Activated Protein Kinase
MGP: Extracellular Matrix Gla Protein
ML: Machine Learning
MMP9: Matrix Metallopeptidase 9
MS: Mayo Score
NF-kB: Nuclear Factor Kappa B
NK: Natural Killers
NLRP3: NLR family pyrin domain containing 3
PBMCs: peripheral blood mononuclear cells
PGA: Physician´s Global Assessment
PMC: Peripheral Mononuclear Cells
PPARG: Peroxisome Proliferator-Activated Receptor Gamma
RB: Rectal Bleeding
rCCR: Regularized Canonical Correlation
ROS: Reactive Oxygen Species
S100A8: S100 Calcium Binding Protein A8
S100A9: S100 Calcium Binding Protein A9
scRNAseq: Single Cell RNA sequencing
SF: Stool Frequency
SNP: Single Nucleotide Polymorphism
TAC: Transit Amplifying Cells
Tfh: Follicular Helper CD4⁺
Tfr: to follicular regulatory CD4⁺
TLR4: Toll-like receptor 4
TMnet: Text Mining Network
TNF-α: Tumor Necrosis Factor Alpha
TOFA: Tofacitinib
TPM: Transcripts Per Million
U: Unmethylated
UC: Ulcerative Colitis
UCdmap: Ulcerative Colitis Disease Map
VEDO: Vedolizumab.
