# Supplementary material for: From tissue to blood: an integrated multi-omics signature identifies fibrogenesis and neutrophil activation as key drivers of ulcerative colitis severity
Source: Front Immunol. 2026 Jul 21;17:1884480. doi: 10.3389/fimmu.2026.1884480 (PMC13433763; doi:10.3389/fimmu.2026.1884480)
Supplement: Supplementary file 1 [file Table1.docx]

**Supplementary Table 1. Cell type deconvolution in blood.** The table contains the results of the non-univariate comparisons between severity stages. For each of them the median and the first and third quartile are displayed. The overall p-value corresponds to the unadjusted Kruskal-Wallis omnibus p-value, while the adjusted overall p-value column corresponds to the false discovery rate of the overall p-value. The remaining columns display the p-values resulting from the pairwise comparisons, computed using the Mann-Whitney test and adjusted using the Benjamini and Hochberg methodology.

| **Cell type** | **Mild** | **Moderate** | **Severe** | **Overall p-value** | **Adjusted Overall p-value** | **Mild**  **vs Moderate** | **Mild vs Severe** | | **Moderate vs**  **Severe** |
| --- | --- | --- | --- | --- | --- | --- | --- | --- | --- |
| **Memory CD4 T cells** | 31.4 [30.4;32.4] | 29.4 [28.4;32.4] | 28.4 [27.4;30.9] | 0.240 | 0.384 | 0.532 | 0.29 | 0.29 | |
| **Naive CD4 T cells** | 16.7 [15.7;18.6] | 16.7 [14.7;17.6] | 14.7 [12.8;15.7] | 0.011 | 0.088 | 0.309 | 0.019 | 0.019 | |
| **Memory CD8 T cells** | 4.90 [1.96;4.90] | 2.94 [1.96;4.90] | 3.92 [1.96;6.37] | 0.726 | 0.774 | 0.844 | 0.794 | 0.794 | |
| **Naive CD8 T cells** | 9.80 [7.84;9.80] | 9.80 [7.84;10.8] | 7.84 [6.86;9.80] | 0.374 | 0.498 | 0.567 | 0.567 | 0.508 | |
| **Mucosal associated invariant T cells** | 4.90 [4.90;5.88] | 3.92 [3.92;4.90] | 4.90 [3.92;4.90] | 0.163 | 0.380 | 0.144 | 0.144 | 0.918 | |
| **ϒδ Vδ2 T**  **cells** | 0.00 [0.00;0.00] | 0.00 [0.00;0.00] | 0.00 [0.00;0.98] | 0.405 | 0.498 | 0.941 | 0.436 | 0.436 | |
| **Memory B cells** | 6.86 [6.86;7.84] | 6.86 [6.86;7.84] | 7.84 [7.84;8.82] | 0.174 | 0.380 | 0.842 | 0.296 | 0.191 | |
| **Naive B cells** | 4.90 [4.90;5.88] | 6.86 [5.88;9.80] | 5.88 [4.90;6.37] | 0.036 | 0.144 | 0.052 | 0.48 | 0.12 | |
| **Classical monocytes** | 8.82 [6.86;11.8] | 8.82 [6.86;9.80] | 11.8 [7.84;14.2] | 0.166 | 0.380 | 0.642 | 0.251 | 0.228 | |
| **Intermidiate non-classical monocytes** | 1.96 [1.96;2.94] | 1.96 [1.96;1.96] | 1.96 [1.96;2.94] | 0.364 | 0.498 | 0.486 | 0.93 | 0.482 | |
| **Neutrophiles** | 0.98 [0.98;1.96] | 0.98 [0.98;1.96] | 1.96 [1.96;2.94] | 0.002 | 0.032 | 0.851 | 0.004 | 0.004 | |
| **Basophils** | 0.98 [0.98;1.96] | 0.98 [0.98;1.96] | 1.96 [1.96;2.45] | 0.023 | 0.123 | 0.527 | 0.152 | 0.019 | |
| **Natural killers** | 1.96 [1.96;1.96] | 0.98 [0.98;1.96] | 1.96 [0.98;1.96] | 0.214 | 0.380 | 0.273 | 0.399 | 0.399 | |
| **Plasmocytoid dendritic cells** | 0.00 [0.00;0.98] | 0.00 [0.00;0.98] | 0.00 [0.00;0.98] | 0.973 | 0.973 | 0.965 | 0.965 | 0.965 | |
