# Supplementary material for: From tissue to blood: an integrated multi-omics signature identifies fibrogenesis and neutrophil activation as key drivers of ulcerative colitis severity
Source: Front Immunol. 2026 Jul 21;17:1884480. doi: 10.3389/fimmu.2026.1884480 (PMC13433763; doi:10.3389/fimmu.2026.1884480)
Supplement: Supplementary file 2 [file Table2.docx]

**Supplementary Table 2. Cell type deconvolution in biopsy.** The table contains the results of the non-univariate comparisons between severity stages. For each of them the median and the first and third quartile are displayed. The overall p-value corresponds to the unadjusted Kruskal-Wallis p-value, while the adjusted overall p-value column corresponds to the false discovery rate of the overall p-value. The remaining columns display the p-values resulting from the pairwise comparisons, computed using the Mann-Whitney test and adjusted using the Benjamini and Hochberg methodology.

| **Cell type** | **Mild** | **Moderate** | **Severe** | **Overall p-value** | **Adjusted Overall p-value** | **Mild**  **vs Moderate** | **Mild vs Severe** | **Moderate vs**  **Severe** |
| --- | --- | --- | --- | --- | --- | --- | --- | --- |
| **Immature enterocytes** | 17.7 [13.7;22.6] | 14.7 [12.7;17.2] | 15.2 [12.5;18.6] | 0.094 | 0.182 | 0.113 | 0.181 | 0.807 |
| **Transit amplyfing cells** | 16.7 [14.7;18.6] | 17.7 [15.7;19.1] | 17.2 [14.5;17.9] | 0.353 | 0.492 | 0.334 | 0.805 | 0.334 |
| **M cells** | 13.7 [12.8;14.7] | 15.7 [14.7;16.7] | 14.7 [13.7;17.6] | 0.016 | 0.090 | 0.013 | 0.107 | 0.516 |
| **Epithelial cells UC** | 14.7 [12.8;16.7] | 12.8 [10.8;14.7] | 15.2 [11.5;18.9] | 0.353 | 0.492 | 0.368 | 0.611 | 0.368 |
| **Tuft cells** | 9.80 [9.80;10.8] | 11.8 [10.8;11.8] | 11.8 [9.80;12.0] | 0.014 | 0.090 | 0.009 | 0.101 | 0.814 |
| **Enterocyte progenitor cells** | 9.80 [7.84;10.8] | 7.84 [7.84;8.82] | 7.84 [6.86;8.33] | 0.087 | 0.182 | 0.136 | 0.136 | 0.413 |
| **Enteroendocrine cells** | 7.84 [6.86;7.84] | 8.82 [7.84;8.82] | 8.82 [7.84;9.07] | 0.032 | 0.128 | 0.051 | 0.051 | 0.779 |
| **Goblet cells** | 5.88 [5.88;7.84] | 5.88 [5.88;6.86] | 6.86 [5.88;7.11] | 0.588 | 0.629 | 0.662 | 0.9 | 0.662 |
| **Cycling transit amplyfing cells** | 4.90 [3.92;5.88] | 5.88 [4.90;5.88] | 5.88 [3.92;6.86] | 0.44 | 0.518 | 0.421 | 0.421 | 0.781 |
| **Enterocytes** | 3.92 [2.94;4.90] | 3.92 [3.92;4.41] | 3.92 [2.94;4.90] | 0.918 | 0.918 | 0.856 | 0.856 | 0.856 |
| **T cells UC** | 5.88 [5.88;7.84] | 7.84 [5.88;9.80] | 5.88 [5.64;7.11] | 0.092 | 0.182 | 0.221 | 0.488 | 0.104 |
| **Naive CD8 T cells UC** | 5.83 [4.85;5.83] | 5.83 [5.83;6.80] | 5.83 [5.83;6.80] | 0.369 | 0.492 | 0.376 | 0.376 | 0.975 |
| **B cell UC** | 8.82 [6.86;8.82] | 10.8 [9.80;12.8] | 9.31 [7.60;10.8] | 0.018 | 0.090 | 0.015 | 0.329 | 0.17 |
| **Memory B cells UC** | 1.94 [0.97;2.91] | 1.94 [1.94;2.91] | 1.94 [0.97;1.94] | 0.232 | 0.387 | 0.318 | 0.718 | 0.312 |
| **Naive B cells UC** | 0.97 [0.97;1.94] | 1.94 [1.94;2.42] | 0.97 [0.97;1.94] | 0.018 | 0.090 | 0.027 | 0.815 | 0.027 |
| **Natural killers UC** | 6.86 [6.86;7.84] | 7.84 [6.86;8.82] | 7.35 [6.86;8.82] | 0.048 | 0.143 | 0.038 | 0.286 | 0.286 |
| **Macrophage UC** | 2.94 [2.94;3.92] | 3.92 [2.94;3.92] | 3.92 [2.94;3.92] | 0.438 | 0.518 | 0.548 | 0.548 | 0.729 |
