# Supplementary material for: From tissue to blood: an integrated multi-omics signature identifies fibrogenesis and neutrophil activation as key drivers of ulcerative colitis severity
Source: Front Immunol. 2026 Jul 21;17:1884480. doi: 10.3389/fimmu.2026.1884480 (PMC13433763; doi:10.3389/fimmu.2026.1884480)
Supplement: Supplementary file 3 [file Table3.docx]

**Supplementary Table 3. The 32 interactions prioritized after text mining approach.** The source and target columns refer to the first and second terms of the interactions found in the data-driven network. The weight corresponds to the correlation found between them. The UCdmap column shows the common target, included in the UCdmap, shared by the two interaction terms. The two remaining columns include the evidence describing the interactions of the source or the target with the common UCdmap target found by the text mining approach.

| **Source** | **Target** | **Weight** | **Ucdmap** | **TM evidence (source)** | **TM evidence (target)** |
| --- | --- | --- | --- | --- | --- |
| PPARG | LCN2 | -0.50211 | TLR4 | 6 | 1 |
| PPARG | LCN2 | -0.50211 | TNF | 32 | 12 |
| PPARG | LCN2 | -0.50211 | IFNG | 4 | 1 |
| PPARG | LCN2 | -0.50211 | IL1B | 18 | 8 |
| PPARG | LCN2 | -0.50211 | CXCL10 | 2 | 3 |
| PPARG | LCN2 | -0.50211 | IL5 | 1 | 1 |
| PPARG | LCN2 | -0.50211 | IL6 | 20 | 10 |
| PPARG | LCN2 | -0.50211 | MMP9 | 9 | 65 |
| PPARG | LCN2 | -0.50211 | EGR1 | 3 | 3 |
| PPARG | LCN2 | -0.50211 | VCAM1 | 3 | 1 |
| PPARG | LCN2 | -0.50211 | NOS2 | 18 | 2 |
| PPARG | LCN2 | -0.50211 | STAT3 | 5 | 4 |
| PPARG | LCN2 | -0.50211 | NLRP3 | 4 | 6 |
| MCL1 | LCN2 | -0.52128 | IL6 | 2 | 10 |
| MAP3K5 | MAPK14 | -0.50343 | TNF | 7 | 1 |
| RRM2 | S100A8 | 0.545245 | MMP9 | 1 | 5 |
| PCSK6 | S100A8 | 0.530465 | MMP9 | 1 | 5 |
| FOS | S100A8 | 0.559513 | MMP9 | 4 | 5 |
| PCSK6 | S100A9 | 0.554076 | MMP9 | 1 | 6 |
| FOSB | S100A9 | 0.506441 | MMP9 | 1 | 6 |
| RRM2 | S100A9 | 0.569525 | MMP9 | 1 | 6 |
| FOS | S100A9 | 0.584443 | MMP9 | 4 | 6 |
| MMP8 | TRIM44 | 0.535303 | MMP9 | 3 | 2 |
| MMP9 | TRIM44 | 0.506418 | TLR4 | 4 | 3 |
| MMP9 | TRIM44 | 0.506418 | CDH1 | 46 | 2 |
| MMP9 | TRIM44 | 0.506418 | MMP9 | 196 | 2 |
| MMP9 | TRIM44 | 0.506418 | RELA | 18 | 1 |
| FOS | IL1RN | 0.53846 | IFNG | 1 | 5 |
| FOS | IL1RN | 0.53846 | MMP9 | 4 | 1 |
| FOS | IL1RN | 0.53846 | IL17A | 1 | 3 |
| PPARG | MAPK14 | -0.50903 | NFKB1 | 3 | 1 |
| PPARG | MAPK14 | -0.50903 | RELA | 7 | 1 |
