# Supplementary material for: From tissue to blood: an integrated multi-omics signature identifies fibrogenesis and neutrophil activation as key drivers of ulcerative colitis severity
Source: Front Immunol. 2026 Jul 21;17:1884480. doi: 10.3389/fimmu.2026.1884480 (PMC13433763; doi:10.3389/fimmu.2026.1884480)
Supplement: Supplementary file 4 [file Table5.docx]

**Validation analysis of data-driven interactions in an independent cohort**

The current file includes the validation analysis for the two mechanisms discussed in our study. Gene expression data from paired blood and colonic tissue samples was retrieved from the following study [1]. To our best knowledge, there is no available dataset consisting of gene expression and DNA methylation data from paired blood and colonic tissue samples from UC patients. Therefore, the validation of the mechanisms suggested was performed using gene expression data only, and assuming a downregulation effect of DNA methylation over PPARG expression. Correlations among the features were used to evaluate whether the same trends as those described in the manuscript could be reproduced using the independent validation data from [1].

The first mechanism discussed in the study involved DNA methylation levels of PPARG in peripheral blood and LCN2 and TLR4 expression values in colonic tissue. Our data showed a negative association of DNA methylation levels of PPARG in peripheral blood mononuclear cells (PBMCs) and expression levels of LCN2 in colonic tissue. Therefore, assuming an inhibitory effect of DNA methylation over gene expression, positive associations between gene expression values of PPARG in circulation and LCN2 in colonic tissue would be expected. In addition, the mechanism suggested that the above-described positive association was related with TLR4 stimulation. This effect could be reproduced using data from [1], since positive correlations among colonic LCN2 and TLR4 and circulating PPARG expression values were observed (Figure 1 of this document). Furthermore, the correlations among the three markers seemed to increase with disease severity.

**Figure 1. 3D Scatter plot of colonic LCN2 and TLR4 expression levels and circulating PPARG expression levels.** Details on data pre-processing can be found in [1].

The second mechanism discussed in the study comprised DNA methylation levels of PPARG and MMP9 expression levels in peripheral blood, along with LCN2 expression values in colonic tissue. Once again, the hypothesis-driving interaction was the negative association observed between DNA methylation levels of PPARG in PBMCs and LCN2 expression in colonic tissue. In this case, the mechanism suggested the formation of the MMP9-LCN2 complex in combination with high expression rate of PPARG. This effect was observed using data from [1], since positive correlations among the three candidates were observed (Figure 2 of this document). Like in the previous analysis, the correlations were higher at more severe stages of the disease, aligning with the results described in the manuscript.

**Figure 2. 3D Scatter plot of colonic LCN2 expression levels and circulating PPARG and MMP9 expression levels.** Details on data pre-processing can be found in [1].

Since this hypothesized mechanism suggests the direct interaction between colonic LCN2 and circulating MMP9 to form the LCN2-MMP9 complex, a direct correlation between these two candidates was computed. This analysis showed a significant correlation between them (p-value < 0.05, Figure 3 of this document). Moreover, the correlation between the two features followed a positive direction, agreeing with the results obtained in our study. Finally, the distribution of severity across the regression line – showing correlation values increasing with severity – also agreed with our findings suggesting that this complex might be formed at more severe stages of UC.

**Figure 3. Scatter plot of circulating MMP9 expression and colonic LCN2 expression.** Details on data pre-processing can be found in [1].

References

[1] Argmann C, Hou R, Ungaro RC et al. Biopsy and blood-based molecular biomarker of inflammation in IBD. Gut. 2023 Jul;72(7):1271-1287. doi: 10.1136/gutjnl-2021-326451.
